# Synergistic mechanism of Ag^+^–Zn^2+^ in anti-bacterial activity against *Enterococcus faecalis* and its application against dentin infection

**DOI:** 10.1186/s12951-018-0336-3

**Published:** 2018-01-31

**Authors:** Wei Fan, Qing Sun, Yanyun Li, Franklin R. Tay, Bing Fan

**Affiliations:** 1 The State Key Laboratory Breeding Base of Basic Science of Stomatology (Hubei-MOST) and Key Laboratory of Oral Biomedicine, Ministry of Education, School and Hospital of Stomatology, Wuhan University, Wuhan, People’s Republic of China; 2 Department of Endodontics, The Dental College of Georgia, Augusta University, Augusta, GA USA

**Keywords:** Silver, Zinc, Ion, Antibacterial, Biofilm, Dentin, *E. faecalis*

## Abstract

**Background:**

Ag^+^ and Zn^2+^ have already been used in combinations to obtain both enhanced antibacterial effect and low cytotoxicity. Despite this, it is still unclear how the Zn^2+^ co-works with Ag^+^ in the synergistic antibacterial activity. The main purposes of this study were to investigate the co-work pattern and optimum ratio between Ag^+^ and Zn^2+^ in their synergistic antibacterial activity against *E. faecalis*, the possible mechanisms behind this synergy and the primary application of optimum Ag^+^–Zn^2+^ co-work pattern against the *E. faecalis* biofilm on dentin. A serial of Ag^+^–Zn^2+^ atomic combination ratios were tested on both planktonic and biofilm-resident *E. faecalis* on dentin, their antibacterial efficiency was calculated and optimum ratio determined. And the cytotoxicity of various Ag^+^–Zn^2+^ atomic ratios was tested on MC3T3-E1 Cells. The role of Zn^2+^ in Ag^+^–Zn^2+^co-work was evaluated using a Zn^2+^ pretreatment study and membrane potential—permeability measurement.

**Results:**

The results showed that the synergistically promoted antibacterial effect of Ag^+^–Zn^2+^ combinations was Zn^2+^ amount-dependent with the 1:9 and 1:12 Ag^+^–Zn^2+^ atomic ratios showing the most powerful ability against both planktonic and biofilm-resident *E. faecalis.* This co-work could likely be attributed to the depolarization of *E. faecalis* cell membrane by the addition of Zn^2+^. The cytotoxicity of the Ag^+^–Zn^2+^ atomic ratios of 1:9 and 1:12 was much lower than 2% chlorhexidine.

**Conclusions:**

The Ag^+^–Zn^2+^ atomic ratios of 1:9 and 1:12 demonstrated similar strong ability against *E. faecalis* biofilm on dentin but much lower cytotoxicity than 2% chlorhexidine. New medications containing optimum Ag^+^–Zn^2+^ atomic ratios higher than 1:6, such as 1:9 or 1:12, could be developed against *E. faecalis* infection in root canals of teeth or any other parts of human body.

## Background

Infectious diseases pose a major threat upon the health and well being of humans [1, 2]. The discovery and development of antibiotics in the last century has dramatically changed the outcome of treatment for infectious diseases. However, the development of bacterial resistance against even the most powerful antibiotics is rapidly becoming a major challenge for the application of antibiotics in combating infectious diseases [3–5]. Because of this concern, stringent standards for the prescription of antibiotics have been established to minimize the development of bacteria resistance, particularly in the hospital environment [5, 6].

Antibacterial metal ions, such as silver ion (Ag^+^) and zinc ion (Zn^2+^), possess broad-spectrum antibacterial activities with no extensive development of bacterial resistance [7]. The silver ion has the most potent bactericidal activity among metal ions and has been used to treat bacterial infections for centuries [8–10]. However, the high cytotoxicity and discoloring potential of Ag^+^ severely restricts its broad application in clinical medicine [11, 12]. To overcome this shortcoming, many researchers attempted to use Ag^+^ with other metal ions, in most cases the Zn^2+^, to achieve strong synergistic antibacterial effect and reduction in cytotoxicity [13, 14]. This is because Zn^2+^ is less toxic but also less bactericidal when compared with Ag^+^ [10, 15]. This effort of synergistic use of Ag^+^ and Zn^2+^ for infection control has been proven effective and efficient in biomedical, medical and dental studies [14, 16, 17]. Nevertheless, the mechanism by which Ag^+^ and Zn^2+^ interacts synergistically with each other is not clearly understood. Moreover, the optimum ratio of Ag^+^ and Zn^2+^ to achieve maximal synergistic antibacterial effect has not been established.

*Enterococcus faecalis* (*E. faecalis*) is a gram-positive facultative anaerobe that can cause refractory infections in the digestive system, urinary tract as well as root canals of human teeth [18–20]. Detection of *E. faecalis* in persistent infection of the root canals and around the root apex of teeth ranged from 24 to 77% [20], and is believed to be a major cause for the failure of root canal treatment [20, 21]. *Enterococcus faecalis* develops strong resistance to extremely harsh environments including highly-alkaline pH, nutritional deficiency and many current clinical intra-canal medications, such as calcium hydroxide [22]. This resistance has made the control of *E. faecalis* infection a great challenge for dentists worldwide.

Silver ion and chlorhexidine have been reported to be very effective in the control of *E. faecalis* infection in root canals [23, 24]. However, Ag^+^ possesses high cytotoxicity and discoloring properties when it is in direct contact with the dentin of root canal walls and tissues surrounding the root apex [25]. Chlorhexidine is also cytotoxic to the apical tissues and produces toxic chemicals when it is used with sodium hypochlorite, a commonly used irrigant for canal debridement in root canal therapy [26, 27]. Chemicals containing Zn^2+^ have also been studied as an intracanal medication against *E. faecalis*, and were found to be more biocompatible but less effective in infection control when compared with medications containing Ag^+^ [15, 26]. Studies on the combined use of Ag^+^ and Zn^2+^ reported an improved antibacterial effect in both intracanal and dental implant surface infection control [28, 29]. Nevertheless, the mechanism and optimum ratio of Ag^+^ and Zn^2+^ in their synergistic antibacterial activity are still elusive, especially in dealing with *E. faecalis* infection.

Membrane potential is a key feature of bacteria for maintaining membrane permeability, intracellular ionic balance, organelle function and quorum sensing, which are essential for the survival of bacterial communities [30–32]. Alteration of the bacteria membrane potential (i.e. hyper-polarization or de-polarization) is an important bactericidal mechanism for many antibacterial medications, including antibiotics [33]. Ion channels on the cell membrane regulate the trans-membrane potential, and the ionic strength in the extracellular matrix affects the activity of ion channels, resulting in the alteration of membrane potential [34]. It is not known whether alteration of membrane potential occurs in synergistic antibacterial activities involving Ag^+^–Zn^2+^. Accordingly, the objective of the present study was to investigate the optimum ratio of Ag^+^ and Zn^2+^ against *E. faecalis* biofilms on dentin and the potential mechanism behind this synergy. Silver ions, Zn^2+^ and chlorhexidine were used independently as controls in the study.

## Methods

### Colony-forming unit counting

The antimicrobial activity of Ag^+^, Zn^2+^ or Ag^+^–Zn^2+^ at different atomic ratios was first examined using a colony-forming unit (CFU) counting method. Briefly, for observation of the antibacterial effect of Ag^+^ or Zn^2+^, a 1 mL suspension [1 × 10^3^ CFUs/mL] of *E. faecalis* (ATCC 29212, ATCC, Manassas, VA, USA) in a double-concentrated BHI broth was incubated with 1 mL either 1.6 × 10^−2^, 3.2 × 10^−2^, 6.4 × 10^−2^, 12.8 × 10^−2^, 25.6 × 10^−2^, 51.2 × 10^−2^ or 102.4 × 10^−2^ mg/mL of silver nitrate solution (AgNO_3_) (Sinopharm Chemical Reagent Co. Ltd., Shanghai, China) or 5.6 × 10^−2^, 16.8 × 10^−2^, 33.6 × 10^−2^, 50.4 × 10^−2^ or 67.2 × 10^−2^ mg/mL zinc nitrate hexahydrate solution (Zn(NO_3_)_2_·6H_2_O) (Sinopharm). For observation of the combined antibacterial activity of Ag^+^ and Zn^2+^, mixed solutions containing 3.2 × 10^−2^ mg/mL AgNO_3_ and 5.6 × 10^−2^, 16.8 × 10^−2^, 33.6 × 10^−2^, 50.4 × 10^−2^ or 67.2 × 10^−2^ mg/mL Zn(NO_3_)_2_·6H_2_O were prepared, corresponding to the Ag^+^–Zn^2+^ atomic ratios of 1:1, 1:3, 1:6, 1:9 and 1:12. A 1 mL suspension [1 × 10^3^ CFUs/mL] of *E. faecalis* in a double-concentrated BHI broth was incubated with 1 mL of the aforementioned mixed solutions, respectively. Solutions with only 3.2 × 10^−2^ mg/mL AgNO_3_ or 67.2 × 10^−2^ mg/mL Zn(NO_3_)_2_·6H_2_O were used as controls. All solutions containing Ag^+^ were prepared, stored and operated in the absence of light.

After incubating at 4 °C for 24 h, 10 μL of the inoculum from each group was plated on brain heart infusion (BHI; Land Bridge Technology Co. Ltd., Beijing, China) broth agar (Biosharp, Hirono, Japan) and incubated anaerobically at 37 °C for 24 h. Bacteria inoculum mixed with only sterilized deionized water was included as blank control group for each test. All tests were performed in sextuplicate for each group and CFUs of *E. faecalis* were counted for group comparisons.

### Dynamic growth-curves

A dynamic growth-curve method was used to observe the dynamic antimicrobial effect of Ag^+^, Zn^2+^ or Ag^+^–Zn^2+^ solutions, Briefly, for Ag^+^, a 4 mL suspension [1 × 10^6^ or 1 × 10^8^ CFUs/mL] of *E. faecalis* was incubated with 4 mL of 1.6 × 10^−2^, 3.2 × 10^−2^, 6.4 × 10^−2^, 12.8 × 10^−2^, 25.6 × 10^−2^, 51.2 × 10^−2^ or 102.4 × 10^−2^ mg/mL AgNO_3_ solution at 37 °C. For Zn^2+^, a 4 mL suspension [1 × 10^3^ CFUs/mL] of *E. faecalis* was incubated with 4 mL of 5.6 × 10^−2^, 16.8 × 10^−2^, 33.6 × 10^−2^, 50.4 × 10^−2^ or 67.2 × 10^−2^ mg/mL Zn(NO_3_)_2_·6H_2_O solutions at 37 °C. For Ag^+^–Zn^2+^, mixed solutions containing the same amount of AgNO_3_ and Zn(NO_3_)_2_·6H_2_O as described in CFU counting method were prepared. Then, 4 mL suspension [1 × 10^6^ CFUs/mL] of *E. faecalis* was incubated with 4 mL of the mixed solutions and 67.2 × 10^−2^ mg/mL of Zn(NO_3_)_2_·6H_2_O at 37 °C, respectively. In addition, a similar mixed solution with doubled concentration of Ag^+^ and Zn^2+^ but the same Ag^+^–Zn^2+^ atomic ratio was prepared. Another 4 mL suspension [1 × 10^8^ CFUs/mL] of *E. faecalis* was incubated with 4 mL of this concentrated Ag^+^–Zn^2+^ mixed solution and 134.4 × 10^−2^ mg/mL of Zn(NO_3_)_2_·6H_2_O at 37 °C, respectively. As in the CFU counting method, bacterial suspension was all prepared in a double-concentrated BHI broth before mixing with various AgNO_3_ and/or Zn(NO_3_)_2_·6H_2_O solutions.

At 1, 2, 3, 4 and 5 h (2, 4, 6, 8 and 10 h for the Zn^2+^ only group using 1 × 10^3^ CFUs/mL *E. faecalis*) after incubation, 1 mL of the suspension was retrieved for optical density (OD) measurement at 600 nm using a spectrometer (UV-2401PC, Shimadzu Corp., Japan). Bacteria inoculum mixed with sterilized deionized water was used as the blank control group. For each group, the test was repeated six times. All solutions containing Ag^+^ were prepared, stored and operated in the absence of light.

### Determination of antibacterial efficiency of synergistic Ag^+^–Zn^2+^ against Ag^+^ only

The antibacterial efficiency of Ag^+^–Zn^2+^ synergy was determined by calculating the bactericidal percentage against same amount of Ag^+^ only, using the information derived from the aforementioned CFU counting and growth-curve tests. The bactericidal percentages against Ag^+^ were calculated for each test using the following equation:

$$ Antibacterial \, efficiency \, \left( \% \right) = \left\{ {1 + \left( {1 - \left[ {\frac{{ {\text{CFUs or ODs }}\left( {{\text{Ag}} + {\text{Zn}}} \right)}}{{ {\text{CFUs or ODs}}\left( {\text{Ag only}} \right)}}} \right]} \right) } \right\} \times 100\% $$

### Minimum inhibitory concentration (MIC) and bactericidal concentration (MBC)

Minimum inhibitory concentration and MBC of Ag^+^, Zn^2+^ or Ag^+^–Zn^2+^ at different atomic ratios were determined by a serial microdilution assay. Briefly, for Ag^+^, 25.6 × 10^−2^ mg/mL AgNO_3_ solution was prepared. For Zn^2+^, 537.6 × 10^−2^ mg/mL Zn(NO_3_)_2_·6H_2_O solution was prepared. For Ag^+^–Zn^2+^, Ag^+^–Zn^2+^ solutions (25.6 × 10^−2^ mg/mL AgNO_3_) at different atomic ratios (1:1, 1:3, 1:6, 1:9 or 1:12) were prepared. From these starting solutions, a series of twofold dilutions were made by adding 50 μL suspension [1 × 10^5^ CFUs/mL] of *E. faecalis* in double-concentrated BHI broth into each well of a 96-well plate with 50 μL Ag^+^, Zn^2+^ or Ag^+^–Zn^2+^ solutions at 37 °C for 24 h anaerobically. An optical density measurement was conducted by a micro-plate reader at 600 nm. Wells with the lowest Ag^+^, Zn^2+^ or Ag^+^–Zn^2+^ concentrations showing OD values most close to that of negative controls were determined to have MIC. MBC was determined by inoculating solutions from wells with Ag^+^, Zn^2+^ or Ag^+^–Zn^2+^ concentrations no less than MIC onto BHI agar plates and calculating the number of viable bacteria to be less than 4% of original bacteria. The test was repeated for three times.

### Cytotoxicity of Ag^+^–Zn^2+^ combinations

To test the cytotoxicity of Ag^+^, Zn^2+^ or Ag^+^–Zn^2+^ combined antibacterial solutions, the cell counting kit-8 (CCK-8) (Dojindo Laboratories, Kumamato, Japan) was used on MC3T3-E1 Cells (ATCC) according to manufacturer’s instructions. Briefly, 5 × 10^3^ cells suspension in 100 μL α-MEM (Thermo Scientific, Waltham, MA, USA) supplemented with 10% fetal bovine serum (FBS) and 1% penicillin/streptomycin (Thermo Scientific) were seeded in each well of a 96-well plate. After incubating at 37 °C with 5% CO_2_ for 24 h, the medium was replaced with 100 μL of fresh α-MEM. For testing of Ag^+^ solutions, a 10 μL of 0.8 × 10^−2^, 1.6 × 10^−2^, 3.2 × 10^−2^, 6.4 × 10^−2^, 12.8 × 10^−2^, 25.6 × 10^−2^ or 51.2 × 10^−2^ mg/mL AgNO_3_ solutions was added respectively. Six wells were employed for each group. For Zn^2+^ solutions, 3 × 10^3^ cells suspension in 100 μL of α-MEM supplemented with 10% FBS and 1% penicillin/streptomycin were seeded into each well of a 96-well plate. After the incubation at 37 °C with 5% CO_2_ for 24 h, the medium was replaced with 100 μL of fresh α-MEM. Then, 10 μL of 5.6 × 10^−2^, 16.8 × 10^−2^, 33.6 × 10^−2^, 50.4 × 10^−2^ or 67.2 × 10^−2^ mg/mL Zn(NO_3_)_2_·6H_2_O solutions was added to each well, respectively. The experiments were conducted in sextuplicate.

For Ag^+^–Zn^2+^, 5 × 10^3^ cells suspension in 100 μL α-MEM supplemented with 10% FBS and 1% penicillin/streptomycin were seeded into each well of a 96-well plate. The procedures described for Ag^+^ or Zn^2+^ were subsequently followed. Then, a 10 μL aliquot of Ag^+^–Zn^2+^ solution (3.2 × 10^−2^ mg/mL AgNO_3_) of different atomic ratios (1:1, 1:3, 1:6, 1:9 or 1:12) was added. Cells exposed to only 3.2 × 10^−2^ mg/mL AgNO_3_ or 67.2 × 10^−2^ mg/mL Zn(NO_3_)_2_·6H_2_O were used as controls. Twelve wells were used for each group.

The medium in each well was removed after 24 h. The cells were washed three times with α-MEM and cultured with 100 μL of α-MEM and 10 μL of CCK-8 solution at 37 °C for 4 h. The absorbance at 450 nm was measured using a micro-plate reader (Power Wave XS2, BioTek Instruments, VT, USA). Wells containing only α-MEM were used for background recording. Cells cultured in medium mixed with 10 μL of sterilized deionized water or 2% chlorhexidine were used as control groups.

### Inhibition of *E. faecalis* biofilm formation on dentin by Ag^+^–Zn^2+^

Dentin slices (4 mm wide × 4 mm long × 1 mm thick) were prepared from human extracted wisdom teeth under a protocol approved by the Ethics Committee of School and Hospital of Stomatology, Wuhan University. All dentin slices were cleaned using an ultrasonic bath in deionized water, 5.25% sodium hypochlorite and 17% ethylenediaminetetraacetic acid successively and finally in deionized water. The cleanliness of the dentin surfaces was checked by a field emission scanning electron microscope (FE-SEM; Ultraplus; Zeiss, Oberkochen, Germany). The dentin slices were autoclaved for 20 min at 121 °C in deionized water and incubated anaerobically in BHI broth for 24 h at 37 °C to ensure no bacterial contamination. Sterilized dentin slices were soaked into 3 mL of *E. faecalis* suspension [1 × 10^8^ CFUs/mL] and incubated under anaerobic condition at 37 °C for 4 weeks. Fresh BHI broth was replaced every second day to remove dead cells and to ensure bacterial viability. After incubation, the slices were rinsed with sterile phosphate buffered saline (PBS) to remove floating bacteria and culture medium. Two randomly-selected dentin slices were observed by FE-SEM to verify the presence of *E. faecalis* biofilm on the dentin surfaces.

After inoculation of the dentin slices, 0.15 g of methylcellulose (Aladdin Industrial Corp., Shanghai, China) was mixed with 2 mL of Ag^+^–Zn^2+^ solution (3.2 × 10^−2^ mg/mL AgNO_3_) with atomic ratio of 1:1, 1:3, 1:9 or 1:12 (as described above), or 2% chlorhexidine. The latter was prepared by diluting 20% chlorhexidine (Adamas, Basel, Switzerland) with sterile deionized water. Addition of the methylcellulose enabled gels to be produced from those solutions. Dentin slices with *E. faecalis* biofilms were embedded in the gels and incubated anaerobically at 37 °C for 7 days in a 100% humidity environment. Gels prepared by mixing 2 mL of sterile PBS with 0.15 g methylcellulose were used as the negative control. Gels prepared by mixing 2 mL of 3.2 × 10^−2^ mg/mL AgNO_3_ or 67.2 × 10^−2^ mg/mL Zn(NO_3_)_2_·6H_2_O with 0.15 g methylcellulose were used as positive control groups. One hundred dentin slices were divided randomly into 10 groups (N = 10). After incubation, all slices were gently washed in sterile PBS for five times to remove remnant gels. Four dentin slices of each group were randomly selected to be observed by FE-SEM (sputter-coated with conductive carbon or gold) and the other six slices from each group were immersed in 8 mL of fresh BHI broth at 37 °C. At 2, 4, 6, 8 and 10 h after incubation, 1 mL suspension was retrieved for OD measurement at 600 nm using a spectrometer (UV-2401PC).

### Zn^2+^ pretreatment

A Zn^2+^ pretreatment study was designed to further understand the role of Zn^2+^ in the synergistic antibacterial effect of Ag^+^–Zn^2+^. Briefly, a 10 mL suspension [1 × 10^8^ CFUs/mL] of *E. faecalis* was centrifuged at 8000 rpm for 5 min. After centrifuging, the supernatant was discarded. To remove the remaining culture medium, the bacteria pellet was suspended in 10 mL of sterilized deionized water and re-centrifuged. The supernatant was then discarded. For Zn^2+^ pretreatment, a 10 mL solution of 33.6 × 10^−2^ mg/mL Zn(NO_3_)_2_·6H_2_O was added to the bacteria and incubated for 30 min at 4 °C. Sterilized deionized water (10 mL) was used as negative control. After 30 min, the Zn^2+^ pretreated bacteria and negative control groups were centrifuged; the bacteria pellet obtained from each group was re-suspended in 10 mL of sterilized deionized water and centrifuged once again to remove the remaining Zn^2+^. The OD values of the suspensions were adjusted to 0.4. Then, 5 mL of Zn^2+^-pretreated bacteria suspension was mixed with 5 mL of 3.2 × 10^−2^ mg/mL AgNO_3_ solutions and 10 mL double-concentrated of BHI broth. For control groups, a 5 mL bacteria suspension from the negative control group was mixed with 5 mL of 3.2 × 10^−2^ mg/mL AgNO_3_ solution or sterilized deionized water, together with 10 mL of double-concentrated BHI broth. At 1, 2, 3 and 4 h after incubation at 37 °C, 1 mL of the suspension was retrieved for OD determination. Experiments were conducted in sextuplicate.

### Membrane potential and permeability of *E. faecalis* treated by Zn^2+^

A BacLight bacterial membrane potential kit (Molecular Probes, Invitrogen, Carlsbad, CA, USA) was used with the cell nucleus stain TO-PRO-3 (Molecular Probes) to determine the membrane potential and permeability of *E. faecalis* treated by Zn^2+^ [35]. Briefly, 1 mL suspension [4 × 10^5^ CFUs/mL] of *E. faecalis* was diluted from a concentrated 1 × 10^8^ CFUs/mL long-phase cultured bacteria suspension in BHI broth and mixed with Zn(NO_3_)_2_·6H_2_O solutions (final concentrations: 5.6 × 10^−2^, 16.8 × 10^−2^, 33.6 × 10^−2^, 50.4 × 10^−2^ and 67.2 × 10^−2^ mg/mL). Untreated suspension of *E. faecalis* was used as the control group. Subsequently, 10 μL of 3 mM 3,3′-diethyloxacarbocyanine iodide (DiOC_2_(3)) and 10 μL of 50 μM TO-PRO-3 were added simultaneously and incubated at room temperature for 25 min. A 1 mL untreated suspension of *E. faecalis* incubated previously with 10 μL of 500 μM carbonyl cyanide 3-chlorophenylhydrazone (CCCP) for 30 min was included as the positive control for DiOC_2_(3); CCCP destroys membrane potential by eliminating the proton gradient [36]. Cells boiled at 100 °C for 10 min were used as the positive control for TO-PRO-3.

For each sample, fluorescence was acquired from 10,000 events by a flow cytometer (Beckman Coulter Inc., CA, USA). Signals were acquired with logarithmic amplification. DiOC_2_(3) was excited at 488 nm, and its green fluorescence was detected through a 525 ± 40 nm band-pass filter and red fluorescence was detected through a 690 ± 50 nm band-pass filter [32]. TO-PRO-3 was excited at 638 nm, and its red fluorescence was detected through a 660 ± 20 nm band-pass filter. Mean fluorescence intensity values of each sample were used for analysis. Because the fluorescence of a bacterial cell or clump is size-dependent, membrane potential was determined by the red/green ratio of DiOC_2_(3) and membrane permeability was evaluated by the values of TO-PRO-3 red/DiOC_2_(3) green. For each group, the test was conducted in triplicate.

### Statistical analysis

Statistical analysis was performed using one-way analysis of variance analysis with a post hoc Student–Newman–Keus test. When the normality or equal variance assumption of the data sets was violated, statistical analysis was performed using non-parametric Kruskal–Wallis analysis of variance with a post hoc Dunn test. For data in normal distribution, mean ± standard deviation (SD) was used, while for data violating normality, median ± quartile (i.e. P25-P75) was used. Statistical significance was preset at α = 0.05.

## Results

### Synergistic pattern derived from CFUs counting

**Fig. 1:**
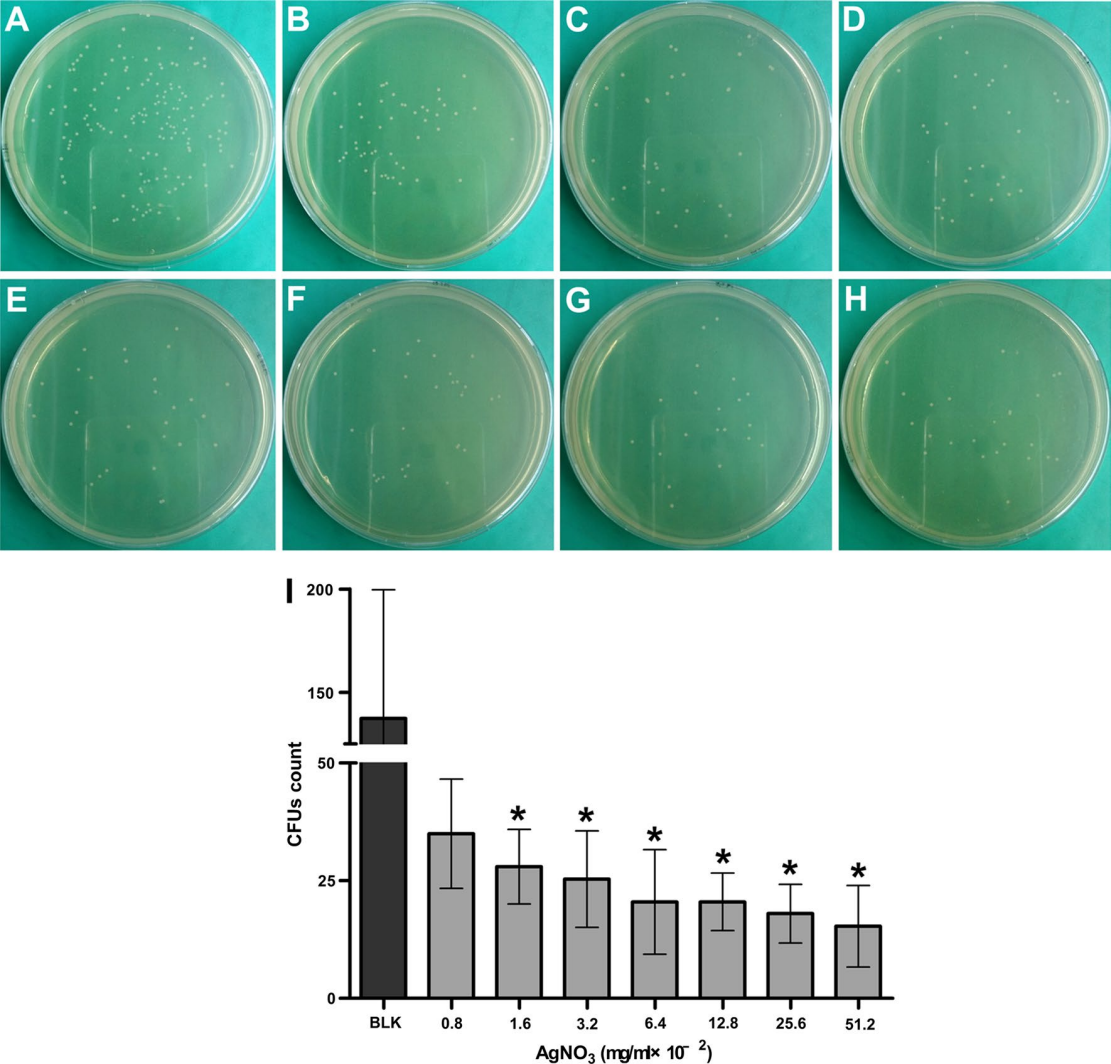
Representative images of bacteria colonies grown on BHI agar showing the antibacterial effect of Ag^+^ (AgNO_3_) against planktonic *E. faecalis*. **A** Blank control group (BLK); **B**–**H** *E. faecalis* incubated with different concentrations of AgNO_3_ (final concentration, in mg/mL, from **B** to **H**: 0.8 × 10^−2^, 1.6 × 10^−2^, 3.2 × 10^−2^, 6.4 × 10^−2^, 12.8 × 10^−2^, 25.6 × 10^−2^, 51.2 × 10^−2^). **I** Comparisons of bacterial CFU count among groups. (Asterisk: significantly different when compared with the blank control group; *p* < 0.05)

**Fig. 2:**
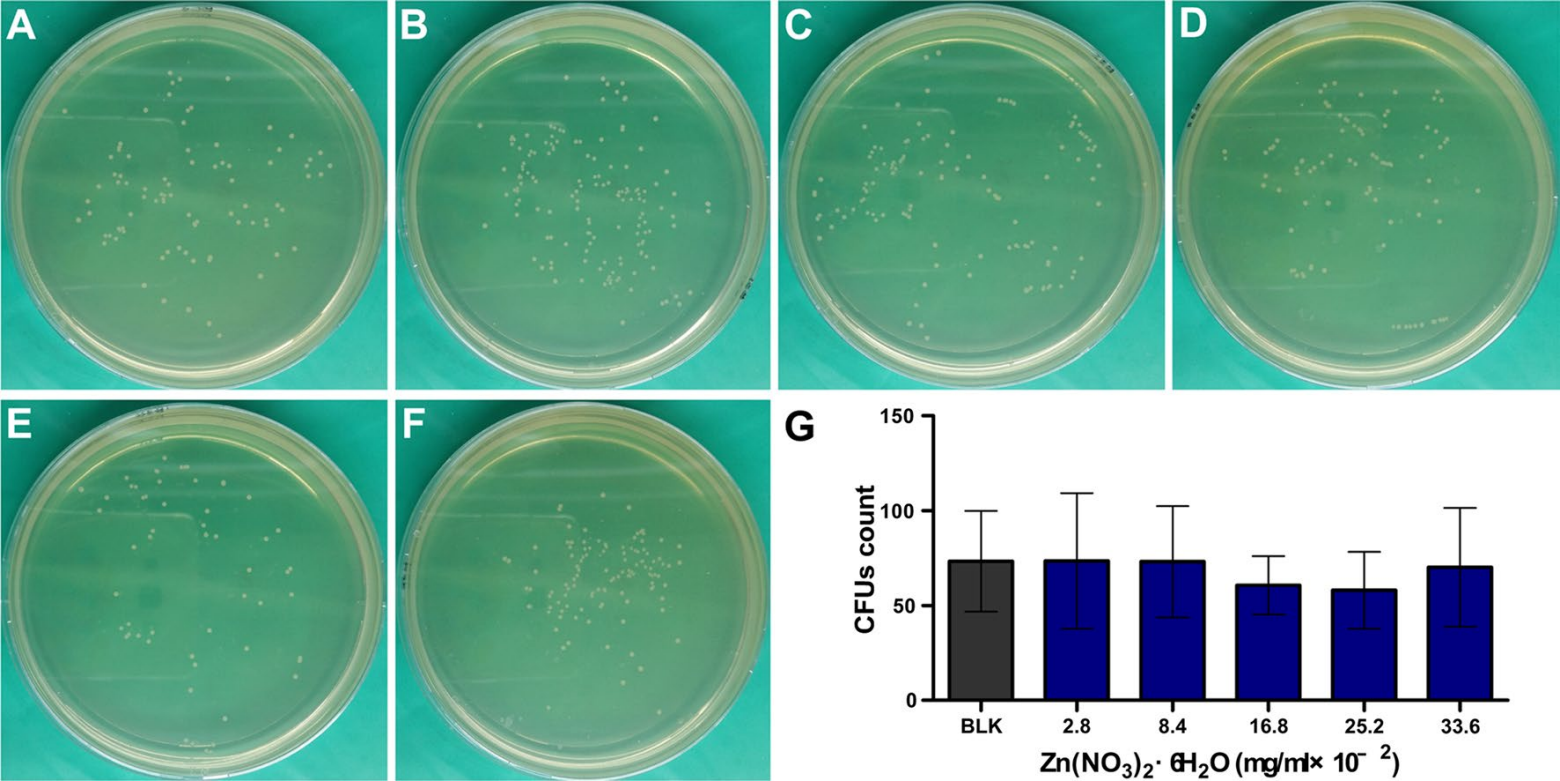
Representative images of bacteria colonies grown on BHI agar showing antibacterial effect of Zn^2+^ (Zn(NO_3_)_2_·6H_2_O) against planktonic *E. faecalis*. **A** blank control group (BLK); **B**–**F** *E. faecalis* incubated with different concentrations of Zn(NO_3_)_2_·6H_2_O (final concentration, in mg/mL, from **B** to **F** 2.8 × 10^−2^, 8.4 × 10^−2^, 16.8 × 10^−2^, 25.2 × 10^−2^, 33.6 × 10^−2^). **G** Comparisons of bacterial CFUs count among groups

**Fig. 3:**
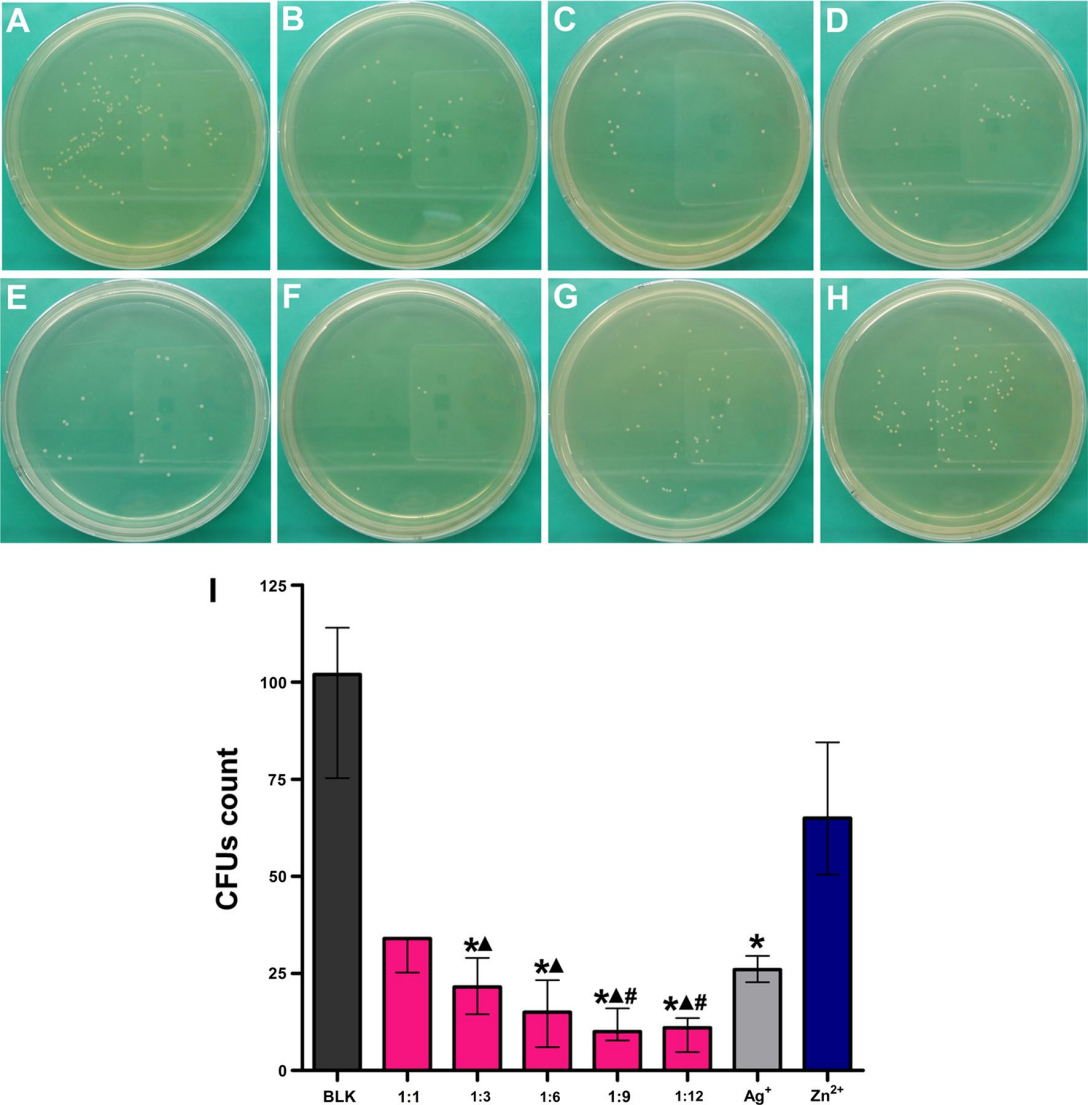
Representative images of bacteria colonies grown on BHI agar showing the antibacterial effect of Ag^+^–Zn^2+^ combinations against planktonic *E. faecalis*. **A** Blank control group (BLK); **B**–**F** *E. faecalis* incubated with different atomic ratio of Ag^+^ and Zn^2+^ (1:1; 1:3; 1:6; 1:9; 1:12); **G** *E. faecalis* incubated with AgNO_3_ only (final concentration, 1.6 × 10^−2^ mg/mL); **H** *E. faecalis* incubated with Zn(NO_3_)_2_·6H_2_O only (final concentration, 33.6 × 10^−2^ mg/mL). **I** Comparison of bacterial CFU counts among groups. (Asterisk: significant difference when compared with BLK; ^▲^significant difference when compared with the Zn^2+^ only group; ^#^significant difference when compared with the Ag^+^ only group; *p* < 0.05)

The CFUs formed on agar plates by *E. faecalis* incubated with Ag^+^ were significantly less than the blank control except the 0.8 × 10^−2^ mg/mL group (*p* < 0.05; Fig. 1). Unlike the bactericidal effect of Ag^+^, no significant difference was observed in the CFUs among the blank control and the various Zn^2+^ group (*p* > 0.05; Fig. 2). There was no decline in CFUs with the increasing Zn^2+^ concentrations. All the Ag^+^–Zn^2+^ groups except the 1:1 group had significantly less CFUs when compared with the blank control and the Zn^2+^ only group (*p* < 0.05; Fig. 3). The 1:9 and 1:12 Ag^+^–Zn^2+^ groups with 1.6 × 10^−2^ mg/mL AgNO_3_ showed significantly better antibacterial effect than the Ag^+^ only group containing 1.6 × 10^−2^ mg/mL AgNO_3_ (*p* < 0.05). The CFUs decreased extensively with increasing amounts of Zn^2+^ in the Ag^+^–Zn^2+^ mixtures.

### Synergistic pattern derived from dynamic growth-curve

**Fig. 4:**
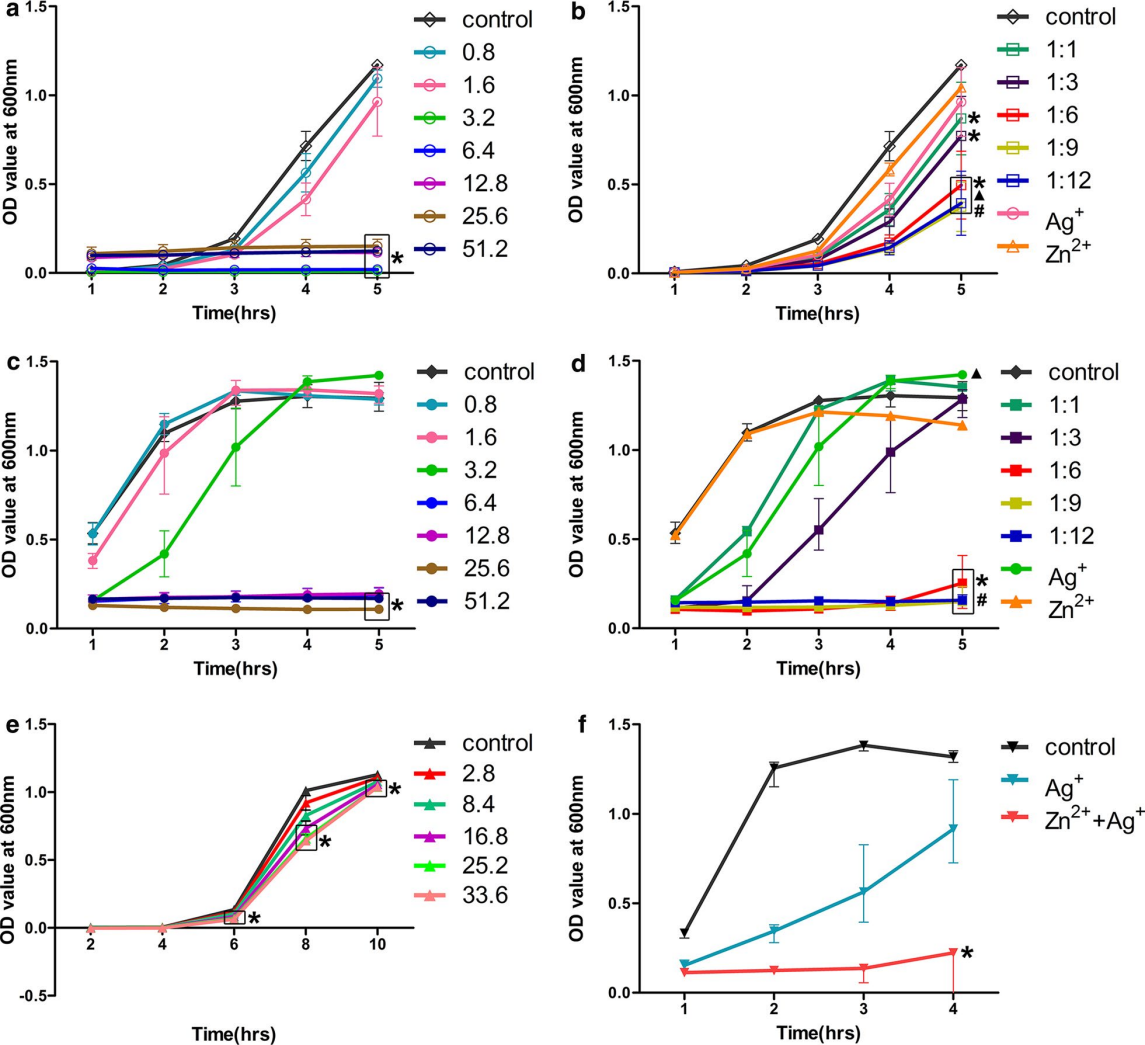
Growth curve of *E. faecalis* under different conditions. *OD* optical density. **a** OD curve of *E. faecalis* (1 × 10^6^ CFUs/mL) incubated with different concentrations of AgNO_3_. **b** OD curve of *E. faecalis* (1 × 10^6^ CFUs/mL) incubated with different Ag^+^–Zn^2+^ ratios. **c** OD curve of *E. faecalis* (1 × 10^8^ CFUs/mL) incubated with different concentrations of AgNO_3_. **d** OD curve of *E. faecalis* (1 × 10^8^ CFUs/mL) incubated with different Ag^+^–Zn^2+^ ratios. **e** OD curve of *E. faecalis* (1 × 10^3^ CFUs/mL) incubated with different concentrations of Zn(NO_3_)_2_·6H_2_O. **f** OD curve of *E. faecalis* (1 × 10^8^ CFUs/mL) pretreated with Zn^2+^ only. (Asterisk: significant difference when compared with the blank control group (*p* < 0.05); ^▲^significant difference when compared with the Zn^2+^ only groups (*p* < 0.05); ^#^significant difference when compared with the Ag^+^ only groups (*p* < 0.05); Groups inside boxes in the corresponding chart had the same labels as the legend

Results from dynamic growth-curve method showed that in Ag^+^ only groups tested with 1 × 10^6^ CFUs/mL *E. faecalis*, groups with AgNO_3_ concentrations of 3.2 × 10^−2^, 6.4 × 10^−2^, 12.8 × 10^−2^, 25.6 × 10^−2^ and 51.2 × 10^−2^ mg/mL had significantly lower OD values than the blank control group (*p* < 0.05; Fig. 4a) at the 5th h. Among the Ag^+^ only groups, those with 3.2 × 10^−2^, 6.4 × 10^−2^, 12.8 × 10^−2^, 25.6 × 10^−2^ and 51.2 × 10^−2^ mg/mL AgNO_3_ showed almost no bacteria growth after 5 h. In Ag^+^ only groups tested with 1 × 10^8^ CFUs/mL *E. faecalis*, groups with 6.4 × 10^−2^, 25.6 × 10^−2^, and 51.2 × 10^−2^ mg/mL AgNO_3_ had significantly lower OD values than the blank control group (*p* < 0.05; Fig. 4c) at the 5th h. No statistical difference with the blank control group was detected in 12.8 × 10^−2^ mg/mL AgNO_3_ group probably due to the relatively low test power of non-parametric statistics despite the similar no bacteria growth after 5 h as 6.4 × 10^−2^, 25.6 × 10^−2^, and 51.2 × 10^−2^ mg/mL AgNO_3_ groups.

In Zn^2+^ only groups tested with 1 × 10^3^ CFUs/mL *E. faecalis*, groups with 8.4 × 10^−2^, 16.8 × 10^−2^, 25.2 × 10^−2^ and 33.6 × 10^−2^ mg/mL Zn(NO_3_)_2_·6H_2_O had significantly lower OD values than the blank control at the 10th h (*p* < 0.05; Fig. 4e).

At the 5th h, in the Ag^+^–Zn^2+^ groups tested with 1 × 10^6^ CFUs/mL *E. faecalis*, groups with 1:1, 1:3, 1:6, 1:9 and 1:12 Ag^+^–Zn^2+^ ratios had significantly lower OD values than the blank control (*p* < 0.05; Fig. 4b). The OD values of 1:6 to 1:12 Ag^+^–Zn^2+^ ratios were significantly lower than the values derived from the Zn^2+^ only group (*p* < 0.05; Fig. 4b). Likewise, OD values of groups with 1:6, 1:9 and 1:12 Ag^+^–Zn^2+^ ratios were significantly lower than values derived from the Ag^+^ only group (*p* < 0.05; Fig. 4b). In the Ag^+^–Zn^2+^ groups tested with 1 × 10^8^ CFUs/mL *E. faecalis*, groups with 1:1 and 1:3 Ag^+^–Zn^2+^ ratios were not significantly different from the blank control (*p* > 0.05; Fig. 4d). Optical density values of groups with 1:6, 1:9 and 1:12 Ag^+^–Zn^2+^ ratios were significantly lower than values derived from the Ag^+^ only group (*p* < 0.05).

### Antibacterial efficiency of Ag^+^–Zn^2+^ against Ag^+^ only

The antibacterial efficiency of Ag^+^–Zn^2+^ combinations calculated using the results derived from CFU counting and growth curve determination are shown in Tables 1 and 2, respectively. In Table 1, antibacterial efficiencies of groups with 1:6, 1:9 and 1:12 Ag^+^–Zn^2+^ ratio were increased by 50.71, 62.28 and 60.05% respectively when compared with the use of only Ag^+^. In Table 2, despite the use of two bacteria concentrations (1 × 10^6^ CFUs/mL and 1 × 10^8^ CFUs/mL), the antibacterial efficiencies of Ag^+^–Zn^2+^ combinations exhibited the same trend as the results presented in Table 1. Antibacterial efficiency of the group with 1:12 Ag^+^–Zn^2+^ ratio was increased by 66.27% (1 × 10^6^ CFUs/mL) and 88.99% (1 × 10^8^ CFUs/mL) when compared with the use of only Ag^+^. Antibacterial efficiency of the group with 1:9 Ag^+^–Zn^2+^ ratio was found very close to that of 1:12 group.

**Table 1:** Antibacterial efficiency of Ag^+^–Zn^2+^ combinations based on CFU counting

Atomic ratio (Ag^+^–Zn^2+^)	AgNO_3_ + Zn(NO_3_)_2_·6H_2_O (× 10^−2^ mg/mL)	Antibacterial efficiency (Median (P25–P75) [%])
1:1	1.6 + 2.8	67.19 (63.37–76.52)
1:3	1.6 + 8.4	108.29 (83.11–146.85)
1:6	1.6 + 16.8	150.71 (118.46–174.25)^a^
1:9	1.6 + 25.2	162.28 (149.30–167.40)^a^
1:12	1.6 + 33.6	160.05 (156.20–174.95)^a^

Groups designated by same superscript are not significantly different (*p* > 0.05)

**Table 2:** Antibacterial efficiency of Ag^+^–Zn^2+^ combinations based on growth curve determination

Atomic ratio (Ag^+^–Zn^2+^)	AgNO_3_ + Zn(NO_3_)_2_·6H_2_O (× 10^−2^ mg/mL)		Antibacterial efficiency (Median (P25–P75) [%])	
	1 × 10^6^ CFUs/mL	1 × 10^8^ CFUs/mL	1 × 10^6^ CFUs/mL	1 × 10^8^ CFUs/mL
1:1	1.6 + 2.8	3.2 + 5.6	111.68 (106.09–113.65)	104.92 (104.10–106.53)
1:3	1.6 + 8.4	3.2 + 16.8	123.58 (113.85–126.03)	109.43 (106.16–115.32)
1:6	1.6 + 16.8	3.2 + 33.6	155.21 (142.46–155.90)^a^	181.98 (172.57–191.55)^a^
1:9	1.6 + 25.2	3.2 + 50.4	165.85 (155.98–167.69)^a^	189.38 (186.85–190.60)^a^
1:12	1.6 + 33.6	3.2 + 67.2	166.27 (150.97–167.80)^a^	188.99 (188.67–189.05)^a^

Groups designated by same superscript are not significantly different (*p* > 0.05)

### MIC and MBC of Ag^+^–Zn^2+^ combinations

Minimum inhibitory concentration and MBC results were shown in Table 3. MIC and MBC of Zn^2+^ were undetectable for its limited antibacterial ability. MIC of Ag^+^ or Ag^+^–Zn^2+^ at different atomic ratios was 6.4 × 10^−2^ mg/mL AgNO_3_. MBC of each group was 12.8 × 10^−2^ mg/mL AgNO_3_ except for the 1:12 group with the MBC being 6.4 × 10^−2^ mg/mL AgNO_3_.

**Table 3:** MIC and MBC values of Ag^+^, Zn^2+^ and Ag^+^ + Zn^2+^ solutions against *E. faecalis*

Groups	AgNO_3_ + Zn(NO_3_)_2_·6H_2_O (× 10^−2^ mg/mL)	
	MIC	MBC_96_
Ag^+^	6.4 + 0	12.8 + 0
Zn^2+^	–	–
Ag^+^–Zn^2+^		
1:1	6.4 + 11.2	12.8 + 22.4
1:3	6.4 + 33.6	12.8 + 67.2
1:6	6.4 + 67.2	12.8 + 134.4
1:9	6.4 + 100.8	12.8 + 201.6
1:12	6.4 + 134.4	6.4 + 134.4

### Cytotoxicity of Ag^+^–Zn^2+^ combinations

**Fig. 5:**
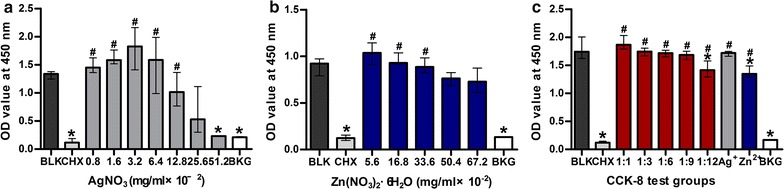
CCK-8 test on cytotoxicity of Ag^+^, Zn^2+^ and Ag^+^–Zn^2+^. **a** MC3T3-E1 cells (5 × 10^3^ cells) exposed to different concentrations of AgNO_3_ (0.8 × 10^−2^, 1.6 × 10^−2^, 3.2 × 10^−2^, 6.4 × 10^−2^, 12.8 × 10^−2^, 25.6 × 10^−2^ and 51.2 × 10^−2^ mg/mL). **b** MC3T3-E1 cells (3 × 10^3^ cells) exposed to different concentration of Zn(NO_3_)_2_·6H_2_O (5.6 × 10^−2^, 16.8 × 10^−2^, 33.6 × 10^−2^, 50.4 × 10^−2^ and 67.2 × 10^−2^ mg/L. **c** MC3T3-E1 cells (5 × 10^3^) exposed to different atomic ratios of Ag^+^–Zn^2+^ (1:1, 1:3, 1:6, 1:9 and 1:12 with 3.2 × 10^−2^ mg/mL AgNO_3_). (*BKG* background, *CHX* chlorhexidine, *BLK* blank control group; Asterisk: significant difference when compared with the blank control group (*p* < 0.05); ^#^significant difference compared with the 2% CHX group. *p* < 0.05)

Results of the CCK-8 tests of Ag^+^ only on MC3T3-E1 cells indicated that 2% chlorhexidine and Ag^+^ only groups with 51.2 × 10^−2^ mg/mL AgNO_3_ had significantly lower cell growth rate when compared with the blank control group (*p* < 0.05; Fig. 5a). When the concentration of AgNO_3_ exceeded 3.2 × 10^−2^ mg/mL, cell growth decreased with the increasing Ag^+^ concentration. No significant difference was detected between the 25.6 × 10^−2^, 51.2 × 10^−2^ mg/mL AgNO_3_ and 2% chlorhexidine (*p* > 0.05; Fig. 5a).

As the cytotoxicity of Zn^2+^ is low, 3 × 10^3^ cells were used for the test to facilitate the observation. It was found that the cytotoxicity of Zn^2+^ increased to the level of 2% chlorhexidine when the concentration of Zn(NO_3_)_2_·6H_2_O reached 50.4 × 10^−2^ mg/mL. No significant difference was detected between the 50.4 × 10^−2^, 67.2 × 10^−2^ mg/mL Zn(NO_3_)_2_·6H_2_O and 2% chlorhexidine (*p* > 0.05; Fig. 5b).

For CCK-8 tests of Ag^+^–Zn^2+^ combinations, all ratio groups except the 1:12 group were not significantly different from the blank control (*p* > 0.05; Fig. 5c). Although the 2% chlorhexidine group, Ag^+^–Zn^2+^ group with 1:12 ratio and Zn^2+^ only groups showed significantly lower cell growth rate when compared with the blank control group (*p* < 0.05), 2% chlorhexidine solutions exhibited the most suppressive effect on cell growth (*p* < 0.05) in all these three groups.

Although the Ag^+^–Zn^2+^ group with 1:12 ratio showed a slight suppressive effect on cell growth, its cytotoxicity was much lower than the 2% chlorhexidine group (*p* < 0.05; Fig. 5c).

### Inhibition of *E. faecalis* biofilm grown on dentin using the optimum Ag^+^–Zn^2+^ ratio

**Fig. 6:**
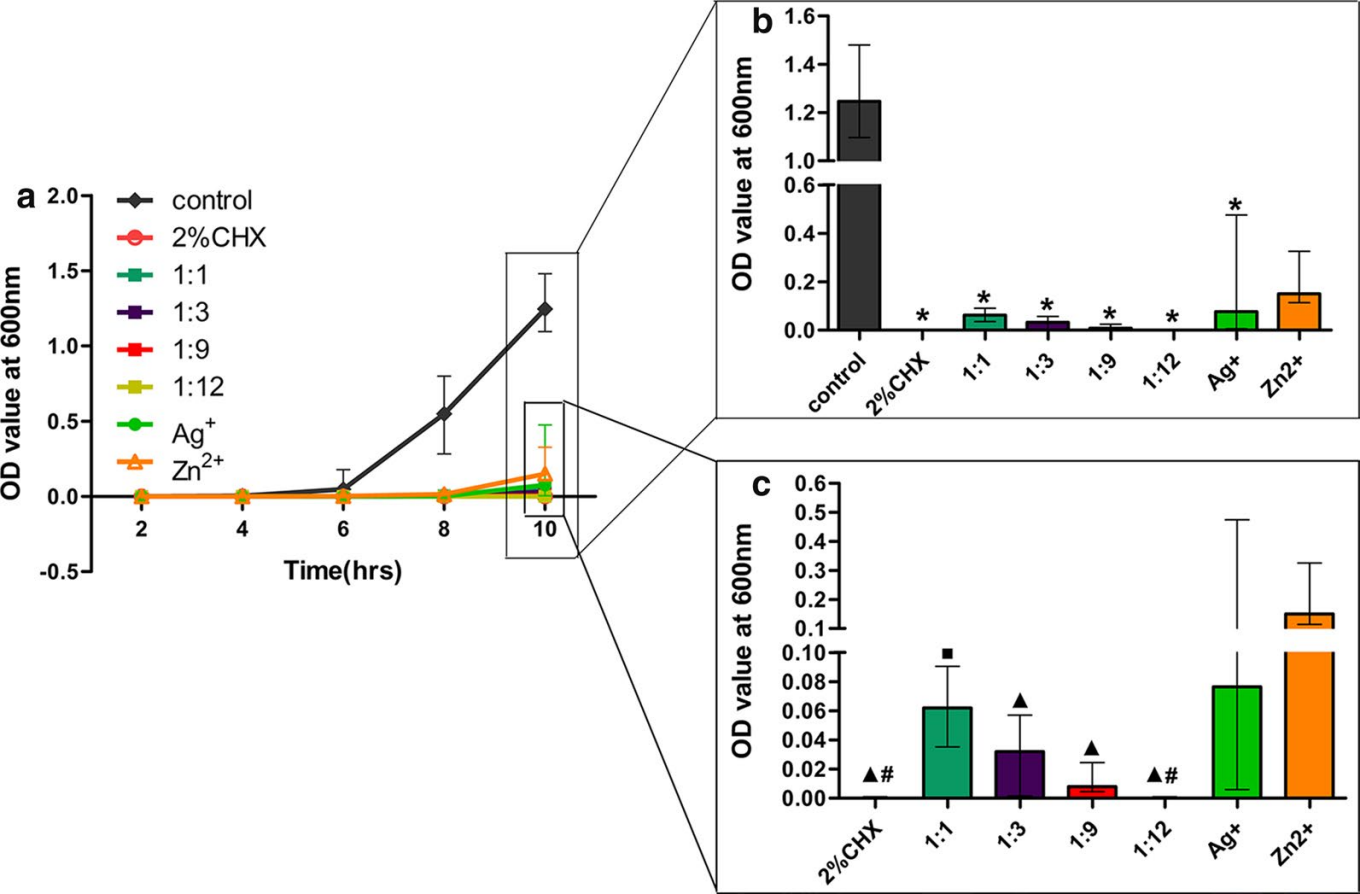
Antibacterial effect of Ag^+^–Zn^2+^ against *E. faecalis* biofilms grown on dentin slices. **a** OD curve within the first 10 h of immersion of dentin slices treated with the different gels for 7 days. **b** Comparisons of the optical density values of each group at the 10th h. **c** Comparisons of the optical density values of each group at the 10th h after exclusion of the blank control group. [Asterisk: significant difference when compared with the blank control group (*p* < 0.05); ^▲^significant difference when compared with the Zn^2+^ only group (*p* < 0.05); ^#^significant difference when compared with the Ag^+^ only group (*p* < 0.05); ^■^significant difference when compared with 2% chlorhexidine (*p* < 0.05)]

**Fig. 7:**
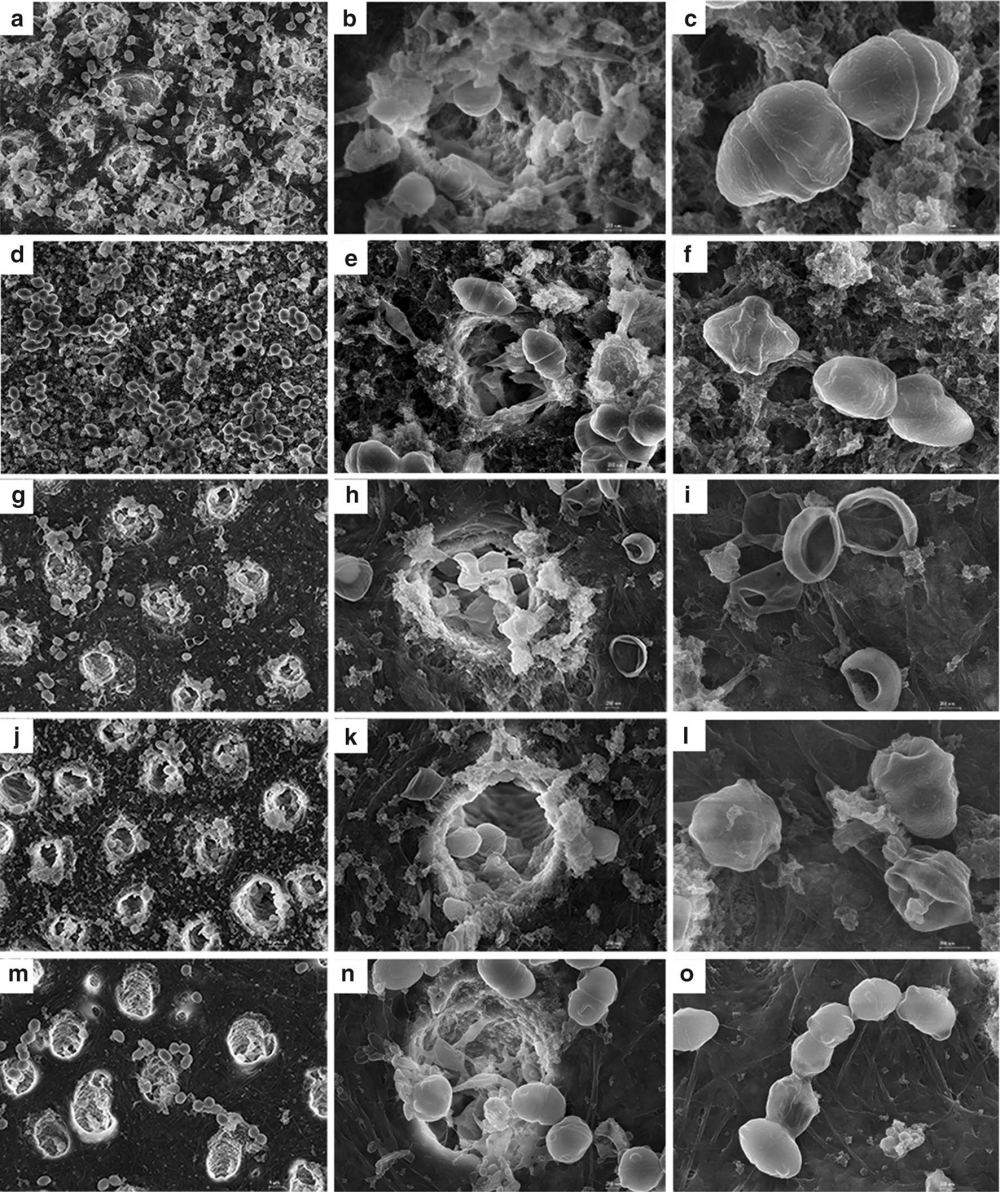
Representative canning electron microscopy images showing the inhibition of *E. faecalis* biofilms grown on dentin. **a**–**c** A biofilm treated with PBS gel for 7 days (**a** ×5000; **b** ×25,000; **c** ×50,000) (negative control). **d**–**f** A biofilm treated with 2% chlorhexidine gel for 7 days (**d** ×5000; **e** ×20,000; **f** ×35,000). **g**–**i** A biofilm treated with Ag^+^–Zn^2+^ gel with 1:12 Ag^+^–Zn^2+^ ratio (3.2 × 10^−2^ mg/mL AgNO_3_) for 7 days (**g** ×5000; **h** ×20,000; **i** ×35,000). **j**–**l** A biofilm treated with gel containing only Ag^+^ (3.2 × 10^−2^ mg/mL AgNO_3_) for 7 days (**j** ×5000; **k** ×20,000; **l** ×50,000). **m**–**o** Biofilm treated with gel containing only Zn^2+^ [67.2 × 10^−2^ mg/mL Zn(NO_3_)_2_·6H_2_O] for 7 days (**m** ×5,000; **n** ×20,000; **o** ×25,000)

Based on the aforementioned findings, the two most potent Ag^+^–Zn^2+^ ratios of 1:9 and 1:12 were used in this anti-biofilm test on dentin. The other two confirmed weaker ratios of 1:1 and 1:3 were also included for comparison. Optical density measurements after direct immersion of dentin slices into fresh BHI solution for 10 h indicated that there was significantly less biofilm accumulation on dentin slices treated with gels containing 2% chlorhexidine, Ag^+^ only or Ag^+^–Zn^2+^ combinations, when compared with the blank control group (*p* < 0.05; Fig. 6b). Dentin slices from the 2% chlorhexidine group and the Ag^+^–Zn^2+^ group with 1:12 ratio had the least biofilm accumulation, especially when compared with the Ag^+^ only group (*p* < 0.05; Fig. 6c). The Ag^+^–Zn^2+^ group with 1:1 ratio showed statistically higher OD value than the 2% chlorhexidine group and the Ag^+^–Zn^2+^ with 1:12 ratio (*p* < 0.05; Fig. 6c). Although no statistical difference was found among 1:3, 1:9, 1:12 and 2% chlorhexidine groups (*p* > 0.05; Fig. 6c), the OD values deceased from 1:3 to 1:12 group with 2% chlorhexidine and 1:12 ratio groups showing the least biofilm accumulation on dentin. Scanning electron microscopy confirmed the differences in biofilm accumulation on dentin slices from different groups, except for those treated with 2% chlorhexidine as 2% chlorhexidine killed and fixed the bacteria biofilm on the dentin surface (Fig. 7) [37].

### Effect of Zn^2+^ pretreatment on the bactericidal activity of Ag^+^

In Zn^2+^ pretreatment study, optical density measurement at the 4th h showed that only the Zn^2+^ pretreatment group had significantly lower OD values when compared with the control group (*p* < 0.05; Fig. 4f). The OD value of Zn^2+^ pretreatment group was also much lower than the Ag^+^ only group despite no statistical difference was detected by the non-parametric statistics in this test.

### The membrane potential and permeability of *E. faecalis* under different Zn^2+^ conditions

**Fig. 8:**
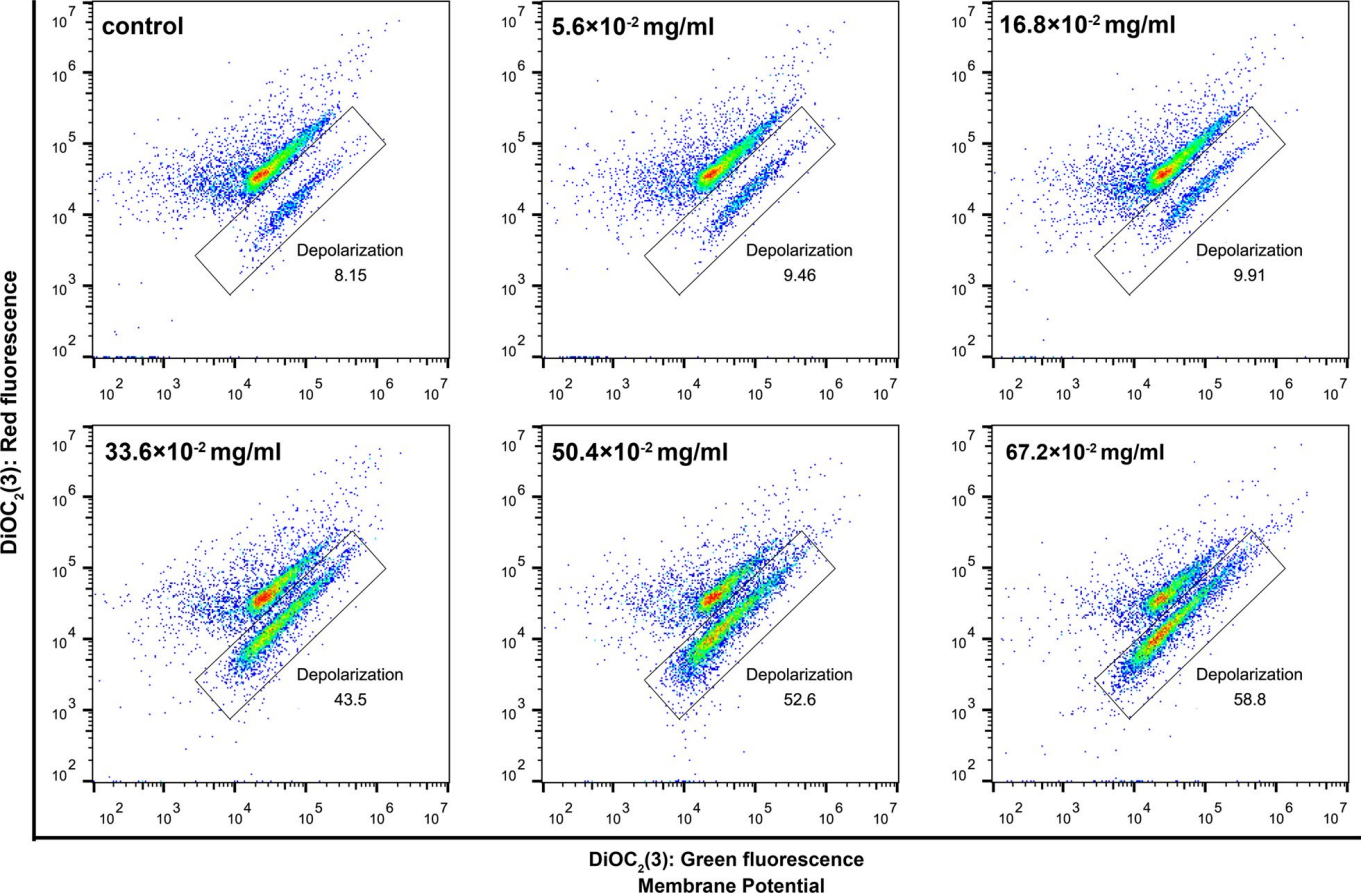
Flow cytometry dot plots showing membrane potential in *E. faecalis* treated with different concentrations of Zn(NO_3_)_2_·6H_2_O. Gates indicate the proportion of depolarized cell population

**Fig. 9:**
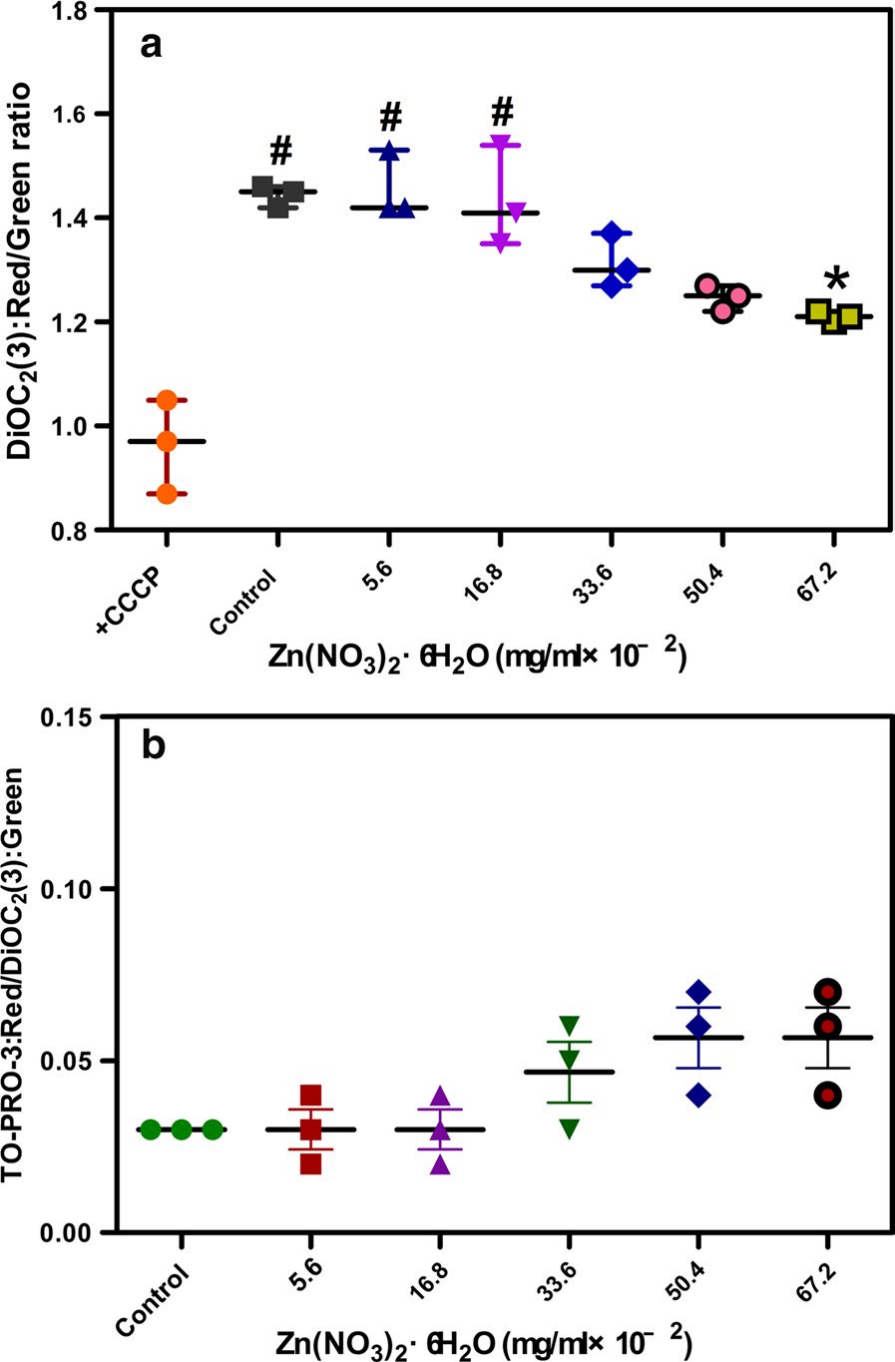
Comparison of membrane potential (**a**) and membrane permeability (**b**) among groups. [Asterisk: significant difference when compared with the blank control group (*p* < 0.05); ^#^significant difference when compared with the CCCP positive control group (*p* < 0.05)]

In the membrane potential and permeability study, the membrane potential of *E. faecalis* exhibited a trend of depolarization with increasing amounts of Zn^2+^ (Figs. 8 and 9a). However, membrane permeability was not altered significantly (*p* > 0.05; Fig. 9b). The largest degree of depolarization of the membrane potential occurred when the concentrations of Zn(NO_3_)_2_·6H_2_O was more than 33.6 × 10^−2^ mg/mL (*p* > 0.05 when compared with CCCP positive control group; Fig. 8).

## Discussion

Silver ions has long since been used as a bactericidal metal element as its strong and broad-spectrum anti-bacterial ability. However, in this study, some *E. faecalis* were found still viable when the AgNO_3_ concentration reached 51.2 × 10^−2^ mg/mL. It might suggest that some strain of *E. faecalis* has developed the ability to resist the bactericidal effect of Ag^+^. On the other hand, the AgNO_3_ concentration higher than 3.2 × 10^−2^ mg/mL exhibited the cytotoxicity. Comparatively, the results from both CFUs counting and growth-curve methods indicated that the bactericidal ability of Zn^2+^ is much weaker than Ag^+^, generally being inhibitive rather than bactericidal to *E. faecalis.* It has been reported that when the Zn^2+^ was mixed with Ag^+^, the anti-bacterial effects would be significantly improved compared to either Ag^+^ or Zn^2+^ alone. However, the pattern and mechanism behind this synergistic activity is still unclear.

In this study, a series of atomic ratios between Ag^+^ and Zn^2+^ was produced by serial dilution, keeping the amount of Ag^+^ constant while increasing the amount of Zn^2+^ in the mixture. Presumably, this helps to evaluate the role contributed by Zn^2+^ in promoting the antibacterial activity of the Ag^+^–Zn^2+^ synergy. Thus, based on the measured MIC of silver nitrate, 1/2 MIC and 1/4 MIC of silver nitrate were selected as the concentration of Ag^+^ in the Ag^+^–Zn^2+^ combinations in order to kept Ag^+^ at a relatively low level (1.6 × 10^−2^ mg/mL AgNO_3_ in which concentration of Ag^+^ is 1.0 × 10^−2^ mg/mL) to facilitate evaluation of its synergistic antibacterial activity with Zn^2+^. Results from the CFU counting method indicated that the synergistic antibacterial effect of Ag^+^–Zn^2+^ combinations is significantly improved only when the Ag^+^–Zn^2+^ ratio is higher than 1:6, i.e. 1:9 and 1:12 in this study. Interestingly, there was no much difference in the antibacterial effect between the 1:9 and 1:12 ratios, which might suggest that these two atomic ratios reach the peak of the synergistic antibacterial ability of Ag^+^–Zn^2+^ combinations. Yang et al. reported a similar phenomenon when 1.7–7% Zn^2+^ was added to 0.3% Ag^+^-doped, heat-resistant honeycomb ceramic materials prepared from red mud industrial waste [13]. However, the combination ratios between the zinc and silver in that study were too limited to identify the mechanism and optimum ratio for Ag^+^–Zn^2+^ synergy against infection. Furthermore, the antibacterial effect of Ag^+^–Zn^2+^ combinations in our study did not show a lower MIC than silver ions only (Table 3), and even the antibacterial effect of 1:12 group was found increased by only 88.99% (Table 2). Considering these findings, it might be more appropriate to use the word “antiseptic” than “antibacterial” in this manuscript. Despite these, these findings could be meaningful in designing new antiseptic or antibacterial materials containing both Ag^+^–Zn^2+^.

The cytotoxicity test revealed that the 1:9 Ag^+^–Zn^2+^ ratios exhibited no suppressive effect on cell growth, while the 1:12 ration showed a slight suppressive effect. Considering the 1:9 ratio having the similar strong antibacterial activity as the 1:12 Ag^+^–Zn^2+^ ratio, the 1:9 Ag^+^–Zn^2+^ ratio might be more appropriate for in vivo applications against *E. faecalis* infection.

In the study against *E. faecalis* biofilm on dentin, 2% chlorhexidine gel was used as the positive control because it is an effective clinical antibacterial agent against *E. faecalis* in root canals [24]. SEM images showed that there seemed still many bacteria left on the dentin surface treated by 2% chlorhexidine, which is different from that of metal ions-treated dentins (Fig. 7). The reason for this difference is because 2% chlorhexidine could kill as well as fix the bacteria biofilm on the dentin surface [37]. The OD values of the Ag^+^–Zn^2+^ group with 1:9 and 1:12 ratios were similar to that of 2% chlorhexidine, which might suggest that Ag^+^–Zn^2+^ combination with a 1:9 or 1:12 atomic ratio was as efficacious as 2% chlorhexidine in eradicating *E. faecalis* biofilms on dentin. Considering that 2% chlorhexidine is significantly more cytotoxic than the Ag^+^–Zn^2+^ combination with 1:9 or 1:12 atomic ratio, the latter may be used to produce new medication against *E. faecalis* biofilm infection on dentin. The bactericidal mechanism of chlorhexidine is different from that of metal ions. Chlorhexidine could kill the bacteria through binding to acidic groups on the surface of the bacteria [38, 39], while metal ions normally enter the cell and combine with the enzyme or other proteins inside the cells, resulting in the cellular malfunctioning and death [40].

To better show and understand how the Ag^+^–Zn^2+^ co-worked in antibacterial activities, a single *E. faecalis* bacteria strain was selected in this study to avoid interferences from different bacteria strains. The synergistic antibacterial effect of Ag^+^–Zn^2+^ combinations could be different on other bacteria or on other materials surfaces with different surface topography [41]. Rathnayake et al. ever reported that multi-drug resistance in clinical isolates (71.2% of *E. faecalis* and 70.3% of *E. faecium*) was much higher than water isolates (only 5.7% *E. faecium*) [42]. Hence, further studies need to be conducted on clinical isolates and multi-species biofilms on different material surfaces, which may express resistance trait against Ag^+^–Zn^2+^ effect.

The Zn^2+^ pretreatment experiment was designed to clarify whether the Zn^2+^ really improves the antibacterial activity of Ag^+^ in the Ag^+^–Zn^2+^ combination. In this experiment, a low Ag^+^ concentration (0.8 × 10^−2^ mg/mL AgNO_3_) was used to facilitate observation of the enhanced antibacterial effect produced by Zn^2+^ pretreatment. The results suggested that Zn^2+^ pretreatment significantly enhanced the sensitivity of *E. faecalis* to the bactericidal activity of Ag^+^. This phenomenon may partly explain why combining Ag^+^ and Zn^2+^ together produces a much stronger antibacterial effect than the use of Ag^+^ alone.

To further investigate how Zn^2+^ makes *E. faecalis* more sensitive to Ag^+^, the membrane potential and permeability of *E. faecalis* was monitored by flow cytometry after the cells were exposed to different amounts of Zn^2+^. This is because membrane potential and permeability are closely related to the sensitivity of bacteria to ionic environment; ion homeostasis affects the proliferation, communication, metabolism and survival of bacteria [30, 34]. Zinc ions have been reported to alter the mitochondria membrane potential of rat brain cells [43, 44]. Nano-zinc oxide, for example, changes the permeability of the cell membrane [45, 46]. The results of this study suggested that the Zn^2+^ depolarizes the membrane potential but does not cause the bacterial cell membrane to rupture and leak. It is consistent with cytotoxicity testing, in which Zn^2+^ showed an inhibitive instead of destructive effect against bacteria. The largest degree of depolarization happened when the concentrations of Zn(NO_3_)_2_·6H_2_O was more than 33.6 × 10^−2^ mg/mL, which might probably explain why the Ag^+^–Zn^2+^ combination was more potent when its atomic ratio was between 1:6 and 1:12.

Maintenance of membrane potential is dependent on normal functioning of the proton pumps and ion channels in the cell membrane [47, 48]. Energy derived from adenosine triphosphate (ATP) is required for homeostasis of membrane potential, including ion transportation through the cell membrane and excretion of toxic heavy metal ions such as Ag^+^ [49]. When the concentration of extracellular Zn^2+^ increases as in the present study, the bacteria will transport some cations, including Na^2+^ and Zn^2+^, into the cell to maintain their membrane potential. However, Zn^2+^ is an inhibitor of ATP synthesis and oxidative phosphorylation in cells [50–52]. P-type ATPases are the essential enzymes involved in bacterial resistance to heavy metal ions [49, 53, 54]. Ion transportation through the cell membrane is hampered when ATP synthesis is inhibited by Zn^2+^. This may result in the accumulation of cations within the cell, including toxic Ag^+^. Intracellular cation accumulation, especially the heavy metal Ag^+^ ions, could result in depolarization of membrane potential and ultimately destruction of cells. The potential pathways through which Zn^2+^ influences Ag^+^ transportation and accumulation requires investigation in future studies.

## Conclusions

Based on the findings of the present study, it may be concluded that the synergistic antibacterial effect of Ag^+^ and Zn^2+^ is dependent upon the amount of Zn^2+^ present in the medium. Atomic Ag^+^–Zn^2+^ ratios higher than 1:6 (i.e. 1:9 and 1:12 in this study) appear to be optimum ratios against both planktonic *E. faecalis* as well as singe-species *E. faecalis* biofilms. This synergistic antibacterial effect may be attributed to the ability of Zn^2+^ to depolarize the bacterial cell membrane. The 1:9 and 1:12 Ag^+^–Zn^2+^ combinations are as efficacious as 2% chlorhexidine against *E. faecalis* biofilms that accumulate on dentin. New medications containing an optimal Ag^+^–Zn^2+^ ratio may be developed against *E. faecalis* infection of tooth root canals as well as infections in other parts of the human body.
